# Asphalt, Where Obtained and How Used

**Published:** 1878-11

**Authors:** 


*ART. III.—Asphalt, where obtained and how used.* By * * * M. D.

This question is so often asked by most intelligent citizens as well as by physicians, myself among the number, without being able to give or receive satisfactory answer, that I have tried to obtain from various sources answer to some of these points of inquiry. As I know little of it except what I obtain from others, it will not be expected that I can write anything original expressing the exact facts of the present time in the practical working and use of this substance, but I find some statements which would seem interesting to every one observing the present uses of this article.

Authors say Asphalt or Asphaltum Bitumen, called also mineral pitch, from the Lacus Asphaltes, or Dead Sea, where it was found in ancient times, is a product of the decomposition of vegetable and animal substances. It is usually found of a black or brownish black color, externally not unlike coal, but it varies in consistency from a bright pitchy condition, with sharp fracture, to thick viscid masses of mineral tar. Asphalt melts at or a little below the boiling point of water, and it burns with smoky flame. It is regarded as the ultimate result of a series of changes in organized matter, producing: 1st, Naphtha; 2d, Petroleum; 3d, Mineral Tar; and 4th, Asphalt or hard bitumen. The whole of these substances merge into each other by insensible degrees, so that it is impossible to say at what point mineral tar ends and asphalt begins.

Naphtha, which is the first of the series, is in some localities found flowing out of the earth, as a clear limpid and colorless fluid; it takes up oxygen from the air, becomes brown and thick and is called in this condition petroleum. A combination of evaporation and oxidation transforms it into mineral tar, and still later into asphalt.

Asphaltic deposits exist widely diffused throughout the world and are very variable in composition, and their proximate constituents have not been subjected to a thorough examination. The most remarkable deposit of Asphalt exists in Trinidad, where it forms a lake 99 acres in extent, and of unknown depth, intersected by rivulets of water. On the lake is seen the emission of semi-fluid tar, still soft and viscid, which, by exposure gradually obtains the consistency of the rest of the mass. In addition to the lake deposit asphalt occurs in the surrounding country in patches or in sheets of considerable size, at one point protruding into the sea, and pieces of asphalt are frequently cast upon the neighboring shore. Asphalt is found also in Cuba, and from Peru a very pure variety of high luster is exported. The asphalt of the Dead Sea is probably a tradition, as very little is found there at the present time. The fountain of Is, on the Euphrates, was the source of supply of ancient Babylon, and still yields asphalt. It occurs in many localities throughout Europe, but not to any considerable extent.

The asphalt beds of the Val de Travers were first discovered and utilized by Eirinus, a Greek Physician, in 1812, who recommended the material as being "peculiarly suitable for covering all kinds of constructions, to protect wood and stone work against decay, worms and the ravages of time, rendering them almost indestructible even when exposed to wind, wet, and extreme variations of temperature." Dr. Eirinus was aware that asphaltic mortar had been used in the building of ancient Babylon, and he himself succeeded in using it with great success in lining cisterns and walls as a cementing material, and for the flooring of warehouses, &c. After some time it fell into disuse and the quarries of Val de Travers were even forgotten until the year 1832, when this material was again re-introduced. Asphaltic stone soon came to be extensively used in France for pavements and roadways, and for protecting floors and walls from the effects of damp. From France the application of the material for such purposes extended to other countries, and there is now a wide spread demand for asphaltic pavements.

It is to be regretted that the present exact process of laying the asphalt pavement cannot be given. I am not aware that the Company or Companies now laying pavements, have yet given their process to the public, nor am I sure that any secrecy is desired. Possibly the Companies would give it in full if requested to do so, or that it has been published and escaped my notice.

Two principal methods are adopted in laying asphalt pavements, the mastic process, and the hot compressed process, as they are called. The mastic process is essentially this: The bed of the roadway is prepared with a smooth level foundation of concrete, which must be thoroughly dry before the application of the asphalt. The mastic is prepared for application by heating the asphaltic stone and breaking it into fine pieces which are then melted up with a quantity of mineral tar, to which sand is added. The molten mass is then poured over a section of the prepared concrete to the requisite depth; the surface smoothed, and covered with a fine sand which is pressed into the asphalt.

The other process now extensively used consists in spreading hot powdered asphalt stone on the prepared surface, which is then heavily pressed till it forms a homogeneous elastic coating.

Roadways so prepared are said to be very durable, smooth, noiseless, cleanly, but not adapted to other than level streets, being slippery and more unsafe than ordinary stone pavements.

An artificial asphalt is prepared by boiling up the pitch of gas-tar with chalk and sand, but such a substitute though cheaper, has not the durability of the natural compound. Gas-tar asphalt is also applied for other purposes in which the natural product is used.

Asphalt was used by the ancient Egyptians in their process of embalming. It is the principal ingredient in black Japan varnish It has also been used for roofing, drain-pipes, water-proof paper and many other purposes.

